# Bright-light distractions and visual performance

**DOI:** 10.3389/fpsyg.2023.1088975

**Published:** 2023-04-25

**Authors:** Craig A. Williamson, Jari J. Morganti, Hannah E. Smithson

**Affiliations:** ^1^Defence Science and Technology Laboratory, Dstl Porton Down, Salisbury, United Kingdom; ^2^Department of Experimental Psychology, University of Oxford, Oxford, United Kingdom

**Keywords:** distractions, visual distractions, visual performance, bright-lights, laser attacks, high dynamic range display

## Abstract

Visual distractions pose a significant risk to transportation safety, with laser attacks against aircraft pilots being a common example. This study used a research-grade High Dynamic Range (HDR) display to produce bright-light distractions for 12 volunteer participants performing a combined visual task across central and peripheral visual fields. The visual scene had an average luminance of 10 cd∙m^−2^ with targets of approximately 0.5° angular size, while the distractions had a maximum luminance of 9,000 cd∙m^−2^ and were 3.6° in size. The dependent variables were the mean fixation duration during task execution (representative of information processing time), and the critical stimulus duration required to support a target level of performance (representative of task efficiency). The experiment found a statistically significant increase in mean fixation duration, rising from 192 ms without distractions to 205 ms with bright-light distractions (*p* = 0.023). This indicates a decrease in visibility of the low contrast targets or an increase in cognitive workload that required greater processing time for each fixation in the presence of the bright-light distractions. Mean critical stimulus duration was not significantly affected by the distraction conditions used in this study. Future experiments are suggested to replicate driving and/or piloting tasks and employ bright-light distractions based on real-world data, and we advocate the use of eye-tracking metrics as sensitive measures of changes in performance.

## 1. Introduction

Distractions can pose a significant risk to transportation safety. Car drivers may have their attention diverted by distractions such as mobile phones or food, with potentially catastrophic consequences (ROSPA, 2020). A year-long study of 100 vehicles found that almost 80% of crashes were caused by distraction or inattention (Neale et al., 2005). Aircraft pilots can be similarly distracted by factors such as non-essential communications and head-down tasks (FAA, 2020). A study of Australian aviation accidents and incidents found that 325 such events over an eight-year period were caused by aircrew distraction (ATSB, 2005). Whether on the ground or in the air, distractions are impairing cognitive performance and disrupting the individual’s ability to perform their primary task of safe transportation (Craik, 2014).

Visual distractions have been shown to reduce the visual field and increase errors in visual tasks. A study of functional visual fields showed that they reduced by as much as 50% when increasing the workload for a central task (Ikeda and Takeuchi, 1975). Another study demonstrated how visual distractors caused a doubling in errors for a peripheral visual task, which also implied a reduction of the useful visual field (Wood et al., 2006). These experiments highlight how some visual distractions can lead to salient information in the periphery being completely missed, with clear implications for hazard detection in a transportation context.

Flashing visual distractions introduce additional challenges by interfering with the eye’s saccadic movements. It has been shown that single or multiple low intensity flashes increase saccadic latency (the time between stimulus presentation and saccade onset), increase target acquisition times, and introduce an error to the initial saccade toward a target (Alvarez et al., 2008). Such results are explained primarily by two concepts: saccadic inhibition, where there is a reduction in the frequency of saccades immediately following a transient visual distraction (Reingold and Stampe, 2002; Buonocore et al., 2016), and inhibition of return, where locations of recent saccades or visual distractions are less likely to be revisited (Abrams and Dobkin, 1994; Wright et al., 2015).

Bright-light visual distractions may also introduce glare (dazzle) into the visual experience. Intense light (such as that from a laser) is scattered within the human eye to spatially spread out the illumination on the retina (Williamson and McLin, 2015). This obscures part of the visual field, with a magnitude that depends primarily on the laser characteristics (power, divergence, range and wavelength) and the eye’s sensitivity adjustments (which are also affected by the ambient luminance; Williamson and McLin, 2018). Laser attacks against aircraft are commonplace (Dietrich, 2017; FAA, 2021), with reported incidents^1^ averaging 50 per year for United Kingdom military aircraft (MOD, 2018), 1,452 per year for United Kingdom commercial aircraft (CAA, 2019), and 7,025 per year for United States commercial aircraft (FAA, 2022).

Previous work has used laser exposures to study the impact of transient bright-light distractions on visual performance. The United States Air Force Research Laboratory (USAFRL) conducted the PEDLS (Performance Evaluation During Laser Strikes) study in 2019 (McLin et al., 2019). That experiment used green laser exposures in the peripheral field of human participants performing a visual task across central and peripheral visual fields. The results showed a reduction in performance in the presence of laser distractions for some conditions, notably those with a low luminance scene and a less challenging visual task. As the laser was directly illuminating participants, this experiment was demonstrating the combined effects of dazzle and distraction. Such combined effects are experienced in an estimated 7% of aviation laser strikes, where laser light enters the cockpit and directly illuminates the eyes of aircrew (Nakagawara et al., 2010).

The present study used a similar experimental paradigm to PEDLS, but presented both the task and the distraction on a High Dynamic Range (HDR) display, and used eye tracking to provide insight into the impacts of the distraction. The intention was to replicate the visual distraction of the PEDLS experiment, but without introducing significant dazzle effects. This represents the remaining 93% of reported laser incidents where the aircrew are not directly illuminated and visual distraction is therefore the primary risk to flight safety. The findings of this study were intended to deepen understanding of bright-light distractions in a transportation context.

The visual task (see section 2.1) combined both a central task and a peripheral visual search task, necessitating multiple fixations across the display area. Two metrics were chosen to assess the impact of bright-light distractions on visual performance: critical stimulus duration and mean fixation duration. The critical stimulus duration for successful task completion was chosen to summarize overall task efficiency in the presence of bright-light distractions. The eye tracking metric of mean fixation duration was chosen to measure participants’ information processing capabilities during the visual tasks. Longer fixation durations permit greater processing time, with more time required to comprehend visual information and process it when cognitive demands are greater (Tole et al., 1982; Irwin, 2004). Fixation durations are typically in the range of 180 to 275 ms for visual search tasks (Rayner, 2009), with an anticipated increase in fixation duration with greater task complexity. The present study’s hypotheses were that bright-light distractions would increase critical stimulus durations (as found in PEDLS with laser distraction). It was also expected that fixation duration would increase in the presence of distractions, and that this eye-tracking metric might provide a more sensitive measure of disruption in visual performance.

## 2. Materials and methods

This study used a delayed match to sample experiment (Macmill and Creelman, 1991) in which participants were required to process visual information across central and peripheral visual fields. The introduction of flashing bright-light distractions to some trials allowed quantification of the impact of these distractions on two dependent variables: mean fixation duration and the critical stimulus duration for successful task completion. Measurement of these variables is explained below.

### 2.1. Visual task

The visual task was adapted from a cognitive “speed training” exercise that was part of the Advanced Cognitive Training for Independent and Vital Elderly (ACTIVE) trial in the late 1990s (Tennstedt and Unverzagt, 2013). This was subsequently evolved into the “Double Decision” brain training game exercise (Brain, 2022), and then adapted by USAFRL for the PEDLS experiment. USAFRL did not formally collaborate on the present study, but they provided digital images and source code from their experiment for re-use and adaptation.

**Figure 1:**
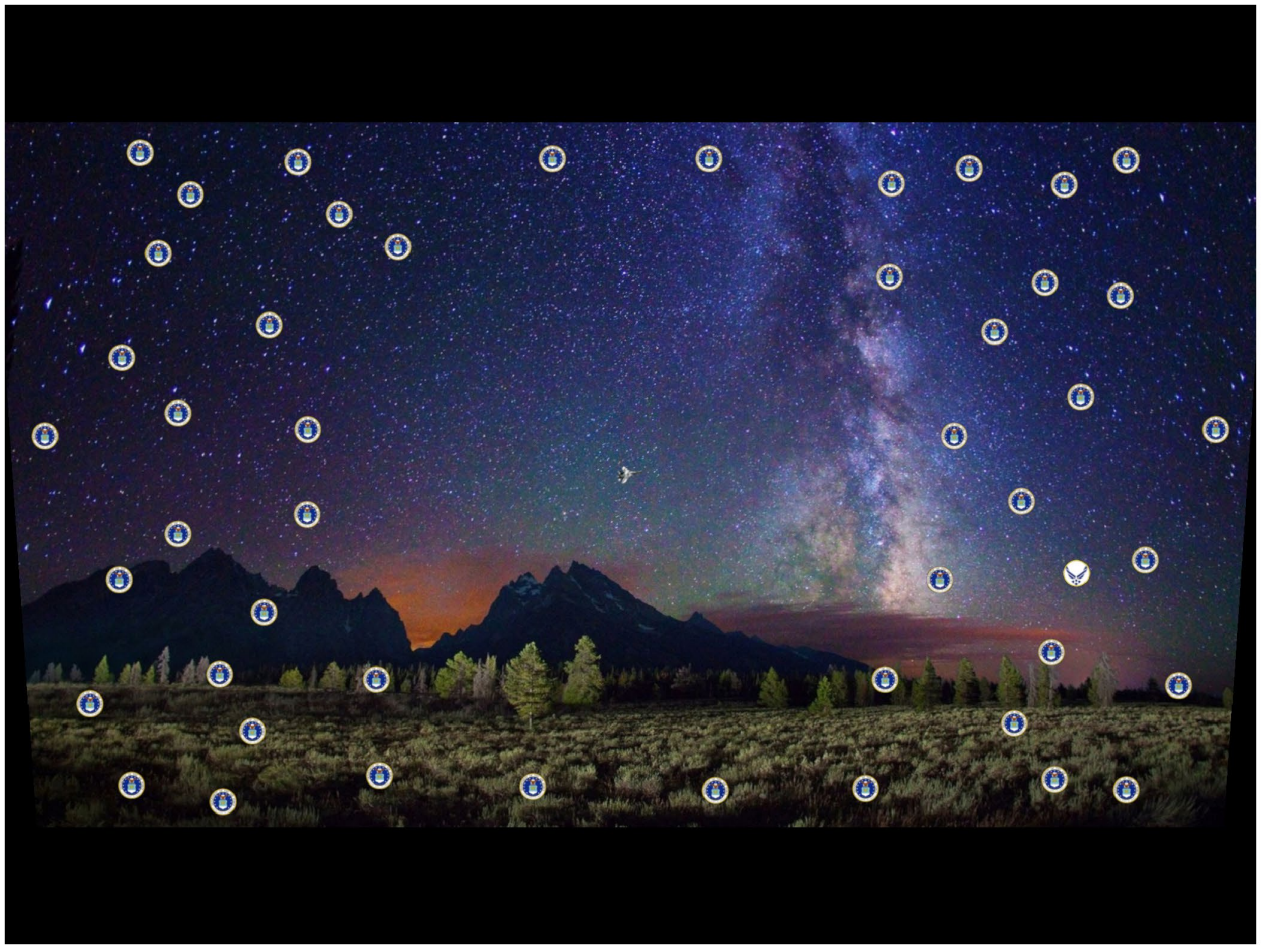
Visual scene shown to the participant.

**Figure 2:**
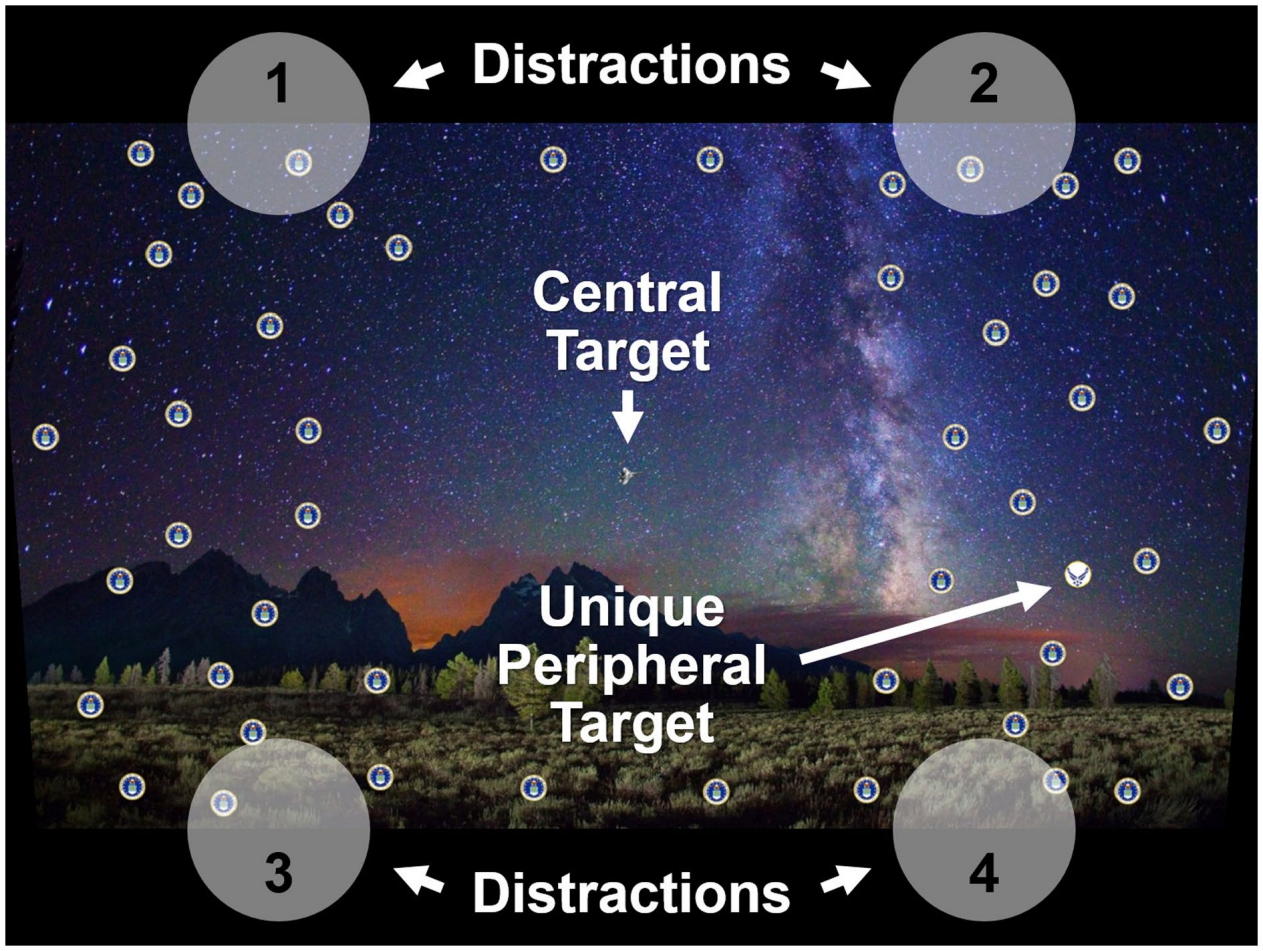
Visual scene shown to the participant, with overlaid annotations to highlight the central target, the unique peripheral target, and the location of the bright-light distractions (1 to 4).

**Figure 3:**
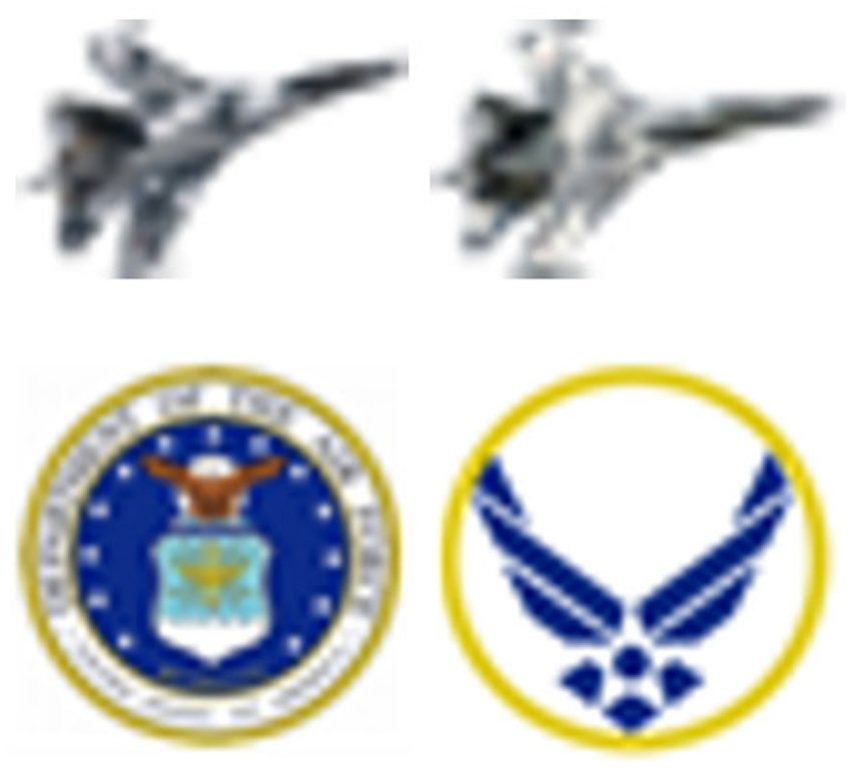
(Top) The two possible central targets. (Bottom) The two peripheral targets: (left) common; (right) unique.

**Figure 4:**
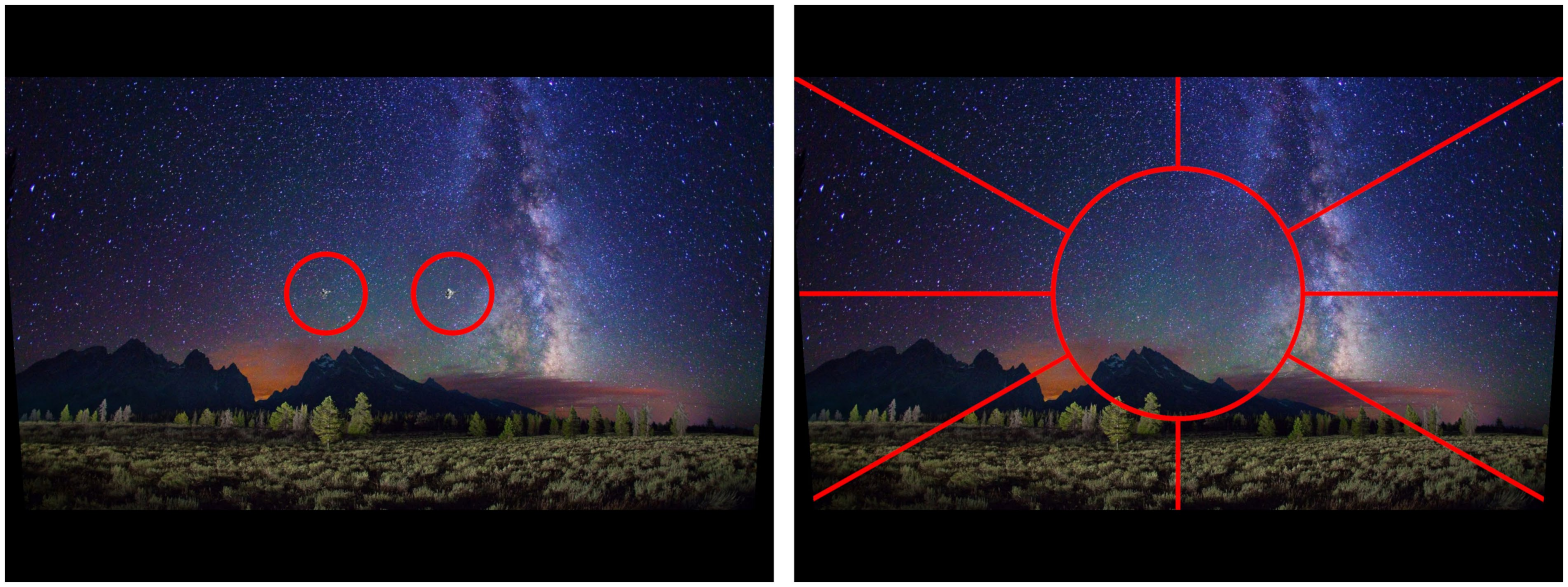
(Left) Selection of central target from two possibilities. (Right) Selection of peripheral target sector from eight possibilities.

Participants viewed a mountain range scene on a display screen, upon which a primary (central) target and a secondary (peripheral) target were displayed simultaneously (Figure 1). The central target was one of two different military aircraft, while the peripheral target was a unique item among 48 distractor items (Figures 2, 3). For each presentation, the choice of central target and the location for the unique peripheral target were randomly generated. The scene was presented to the participant for a time that was determined according to the rules set out below. For the main experiment, presentation duration was adjusted from one presentation to the next in a staircase procedure to find the “critical stimulus duration” required for successful task completion (i.e., one of the two dependent variables measured in this experiment). Participants were free to develop their own visual search strategy to complete the central and peripheral tasks, and no instructions were given on this aspect. Gaze position was recorded with an eye tracker during each stimulus presentation, and fixations were extracted from the eye-tracking data in post-processing to determine “mean fixation duration” (i.e., the second dependent variable). After presentation of the target stimuli, participants were shown an input screen to identify the central target from a choice of two alternatives whose position (left and right) was randomized (Figure 4). The participant used a computer mouse to respond by clicking on which central target they thought was shown, with an auditory tone given to indicate if they were correct (high pitch tone) or incorrect (low pitch tone). They were then shown a grid overlay on the empty scene and again used the mouse to indicate the sector in which the unique peripheral target appeared, from a choice of eight alternatives (Figure 4). Auditory feedback on task performance was given again. The task was deemed to have been completed successfully if both responses were correct. As shown in Figures 1, 4, the same background mountain range scene was displayed throughout the target presentation and the input screens. The images used and the user response methodology were identical to those of the PEDLS experiment in order to allow conclusions to be compared. The parameters of the display and scene are detailed in sections 2.2 and 2.3, and summarized in Table 1.

**Table 1:** Parameters for the display system, and the scene and distraction configuration.

Parameter	Value
Display horizontal viewing distance	45 cm
Display size	197 mm × 148 mm
	2,048 pixels × 1,536 pixels
	24.7° × 18.7°
Central target size	0.5° × 0.4°
Peripheral target size	0.6° × 0.6°
Scene luminance	1–18 cd·m^−2^ (avg. 10 cd·m^−2^)
Low luminance distraction	10 cd·m^−2^
High luminance distraction	4,000–9,000 cd·m^−2^
Distraction circle diameter	300 pixels
	3.6°

After the participant responded to both questions, the display duration for the next presentation was determined using the adaptive threshold algorithm QUEST (Watson and Pelli, 1983). The display duration decreased after a successful identification of the central target and the peripheral target sector location, but increased if there was an error on either task. The threshold display duration for which the QUEST algorithm estimated success at both tasks with a probability of 80% was identified as the “critical stimulus duration.” A sequence of presentations leading to a threshold duration estimate is called a “trial,” and the trial was ended after the minimum of 40 presentations or the number of presentations required to yield a threshold estimate with less than 0.1 s uncertainty at a 90% confidence level. A single trial took around 5 min.

On some trials, participants were exposed to a random flashing distraction that appeared at one of four locations along the outside perimeter of the screen (labeled 1 to 4 in Figure 2). The random distractions consisted of a circular area of pixels on the screen that emitted a solid white color at one of two luminance levels. Only a single luminance level was used in each individual trial. The “low” luminance level was set to match the average luminance of the scene, while the “high” luminance level was set at the maximum achievable luminance of the display for the given average scene luminance (for further details of luminance levels see section 2.3). These two levels were used to elicit the differences between standard visual distractions and “bright-light” visual distractions. The location of the random distraction (1 to 4), its on-state time, and its off-state time changed randomly and continuously throughout each presentation. These times were between 0.1 and 0.5 s for the on-state, and 0.15 to 0.5 s for the off-state, chosen based on pilot testing and information from the USAFRL PEDLS experiment. If the QUEST algorithm requested any presentation durations of less than 0.1 s, they were set to 0.1 s for compatibility with these constraints.

### 2.2. Display system

**Figure 5:**
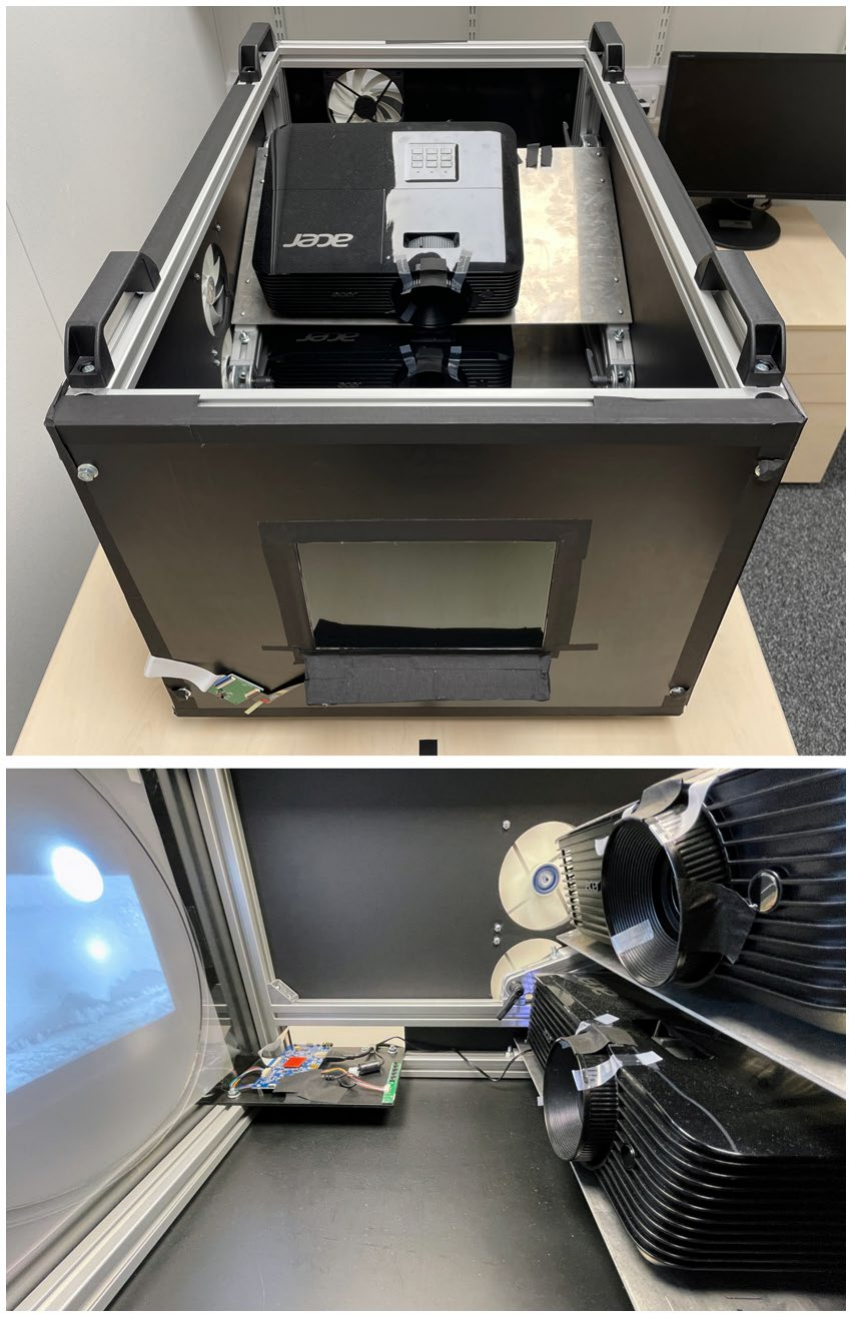
MPHDR display system used in this experiment (top) system with the cover removed (bottom) interior showing control electronics and the two projectors, of which only the lower one was used for this experiment.

The display system used for this experiment was a bespoke Multi-Primary High Dynamic Range (MPHDR) display with two spectrally filtered internal projectors incident upon a transmissive LCD panel taken from an Apple iPad® (Figure 5; Hexley et al., 2020). This was used in its HDR mode, using only the lower of the two internal projectors and removing the spectral filter from the projector output. This permitted luminance levels approaching 10,000 cd∙m^−2^ to be achieved with a dynamic range of approximately 10^5^. The display was geometrically calibrated for the viewing location using a Canon EOS Rebel T1i Digital SLR camera, and spectrally calibrated with a Cambridge Research Systems (CRS) SpectroCAL spectroradiometer and a Konica Minolta LS-110 luminance meter. These calibrations ensured uniformity of the stimuli being displayed to participants.

The viewing location was set at a horizontal distance of 45 cm and an eye height of 10 cm above the vertical center of the display. This slightly downward viewing angle was required for the best uniformity and highest luminance from the display, as the internal projector is angled pointing slightly upwards. A tapered lower crop of the source images was required (Figure 1) to conform to this viewing angle and avoid leak-through of the unfiltered projector luminance. The display size is 197 mm × 148 mm with 2,048 pixels × 1,536 pixels. Viewed at 45 cm, this gave a field of view of 24.7° (horizontal) × 18.7° (vertical). These parameters are summarized in Table 1.

### 2.3. Scene and distraction configuration

Scene and distraction parameters are summarized in Table 1. The angular size of the central targets was approximately 0.5° × 0.4° (around 40 pixels × 30 pixels) and the peripheral targets were approximately 0.6° × 0.6°. The peripheral task required lower visual resolution than the central task due to the nature of the targets.

The luminance of pixels in the scene ranged from 1 to 18 cd∙m^−2^, with an average of around 10 cd∙m^−2^. This level was chosen so that participants were in the photopic regime, but with sufficient dynamic range to provide a large contrast to the high luminance distraction. Two luminance levels were used for the distraction. The “low” luminance distractions were set to match the average scene luminance at around 10 cd∙m^−2^. The “high” luminance distractions were set to the maximum achievable luminance, which was around 4,000 cd∙m^−2^ in locations 1 and 2 (see Figure 2) and around 9,000 cd∙m^−2^ in locations 3 and 4. At less than 10,000 cd∙m^−2^, this was inherently safe according to the guidance of the International Commission on Non-Ionizing Radiation Protection (ICNRP, 1997, 2013).

The distraction for both the “low” and “high” luminance conditions was displayed as a white circle of 300 pixels diameter, centered at one of four locations at the edge of the scene display (Figure 2). While laser incidents are narrowband, with green being the most commonly encountered color (FAA, 2021), white light was used in this experiment to achieve the maximum possible luminance. The Red:Green:Blue luminance ratio of the HDR display was approximately 2:7:1, meaning that using white light gave approximately 40% higher luminance than green. Regarding the distraction size, 300 pixels represents a 3.6° full angle obscuration to the participant. This size was selected based on pilot data, with the intention of providing a clear visual distraction without obscuring many of the peripheral targets. As seen in Figure 2, one peripheral target was obscured in each of the distraction locations.

### 2.4. Implementation

**Figure 6:**
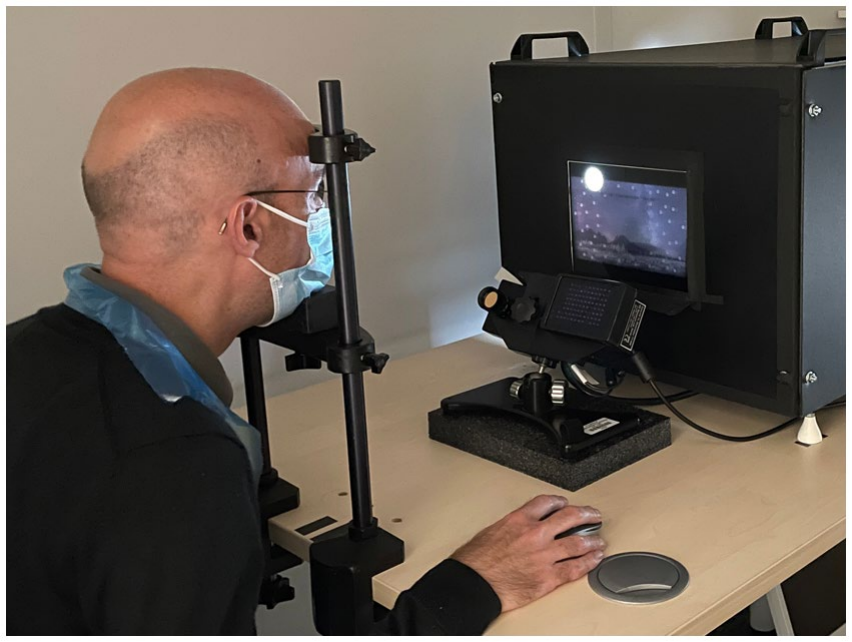
Participant viewing the HDR display and using a mouse to respond to the task. The eye tracker can be seen between the participant and the display. Room lighting was switched off during the experiment. The participant consented to use of their image in this publication.

The experiment was driven by a MATLAB script that utilized the Psychophysics Toolbox extensions known as “Psychtoolbox-3” (Brainard, 1997; Pelli, 1997; Kleiner et al., 2007), together with the Eyelink Toolbox (Cornelissen et al., 2002). Participants viewed the HDR display while sat in an adjustable chair in an unlit room with their head supported by a chin rest and brow pad (Figure 6). The visual scene was viewed monocularly with the right eye, while the left eye was covered with an eye patch. An SR Research EyeLink® 1,000 was used to record eye tracking data (Figure 6).

The experimental design had three levels of distraction (Off, Low or High), four repetitions per session, and two data collection sessions (sessions two and three) plus one practice session (session one). This gave a total of 24 data-collection trials per participant. The first session consisted of four practice trials without distractions in order to build familiarity with the experiment and reduce any learning effects. This same set of four familiarization trials took place at the start of sessions two and three. However, in those two sessions the familiarization trials were followed by 12 data-collection trials in groups of three, with a counterbalanced order of random distractions (e.g., Off–Low–High, then Off–High–Low). The first data collection trial of each repetition was always in the “Off” configuration (i.e., without the random distraction) in order to evaluate the effect of distraction-on trials after baseline distraction-off trials. Each individual trial began with three presentations of a relatively long duration (6, 4, and 3 s) that were not reported to the QUEST algorithm. This was to allow a short acclimation period for participants. The fourth presentation began the official data-collection period with an initial presentation duration of 2 s. Each of the data-collection sessions lasted approximately 90 min.

The within-subjects design of the experiment was to be evaluated with respect to each individual’s mean fixation duration and mean critical stimulus duration across their eight trials in each of the three distraction configurations.

### 2.5. Volunteer cohort

Twelve human volunteers participated (seven females and five males), with a mean age of 20.2 ± 0.7 years and a mean eye (iris) pigmentation of 0.9 ± 0.2 [where eye pigmentation is subjectively quantified as 0 for very dark, 0.5 for dark, 1 for light and 1.2 for very light eyes, with lighter eyes expected to experience greater intraocular scatter (Vos et al., 2002)]. This was the same number of participants as in the USAFRL PEDLS experiment. Eye tracking data were collected for 10 of the 12 participants, with calibration of the eye tracker being unsuccessful with the other two participants. The study was open to participants between the ages of 16 and 65 with normal color vision, normal visual acuity (for two participants this was corrected with spectacles), and no self-reported history of epilepsy, migraines, or hypersensitivity to light. Color vision was assessed using the Fourth Edition of HRR (Hardy-Rand-Rittler) Pseudoisochromatic Plates (Hardy et al., 1954; Dain, 2004). Visual acuity was assessed using a tumbling E (Taylor, 1978) on the HDR display, with a limb size of 5 pixels and a letter size of 25 pixels. The central task only required discrimination of the target from two possibilities, and it was therefore judged to be equivalent to a recognition task that would typically require 4 cycles (line pairs of white/black) to achieve according to the Johnson criteria (Johnson, 1985). This led to a minimum visual acuity requirement for the task to be a minimum angular resolution of 5 pixels (= 3.67 arc minutes for the display system at the viewing distance) at the display scene luminance.

This study was reviewed by, and received ethics clearance through, the University of Oxford Central University Research Ethics Committee (CUREC; R73715/RE002) and the Ministry of Defence Research Ethics Committee (MODREC; 2014/MODREC/21). Informed consent was obtained from all participants prior to their participation. Data collection took place during November 2021 in the Department of Experimental Psychology at the University of Oxford.

### 2.6. Data processing and analysis

Data processing and analysis was conducted using IBM SPSS Statistics for Windows (Version 27.0), MATLAB for Windows (version R2019b), the Edf2Mat MATLAB Toolbox (version 1.20; Etter and Biedermann, 2022), and Microsoft Excel for Windows (version 2016). For analysis of the eye-movement data, periods corresponding to blinks were labeled by the Eyelink parser (SR Research Ltd, 2009) and excluded from the analysis. Fixations were identified, again using the Eyelink software, as periods between saccades (identified from velocity and acceleration profiles). Mean fixation durations were calculated per trial (10 participants with eye tracking data × 3 conditions × 8 repeats). After removal of partial data (e.g., due to eye-tracking failures in a given condition for a given participant), there were 201 mean fixation durations that were fully matched across all test conditions. Critical stimulus durations were also calculated per trial (12 participants × 3 conditions × 8 repeats), with 288 values fully matched across all test conditions. For both dependent variables (mean fixation duration and critical stimulus duration), outliers for a given participant in a given condition for each of the trial repetitions were identified at the ±2 SD level [see marked values in the raw datafiles (Williamson et al., 2022)].

Group-level analysis was performed on data from all available participants, with a separate analysis for each of the dependent variables (mean fixation duration, *n* = 10; and critical stimulus duration, *n* = 12). In the experimental design, each participant contributed data in each of three experimental conditions (distraction Off, Low, High), so a one-way repeated measures ANOVA with three levels was used to test for an effect of distraction type on the dependent variable.

This group-level analysis tests for a general effect across the sampled population. However, it is known that there are large individual differences in susceptibility to distractions during visual search, arising from differences in goal-directed attentional control and from differences in sensitivity to the distractor (Lechak and Leber, 2012). For these reasons, we used the repeated data collected from each participant to first test for differences between participants and the way they were affected by the distractor manipulation (participant by distractor interaction). A significant interaction was explored by performing analyses at the individual participant level using the eight repeats of each of three distractor conditions in a one-way repeated measures ANOVA. Note that, in this secondary analysis, no inferences are made about group-level effects; instead, for a given individual, the test is whether there are reliable differences between distraction types, collectively over repeated measurements.

In all cases, the use of ANOVA was supported by confirmation of normality using the Shapiro–Wilk test and confirmation of sphericity using Mauchly’s Test of Sphericity.

## 3. Results

**Figure 7:**
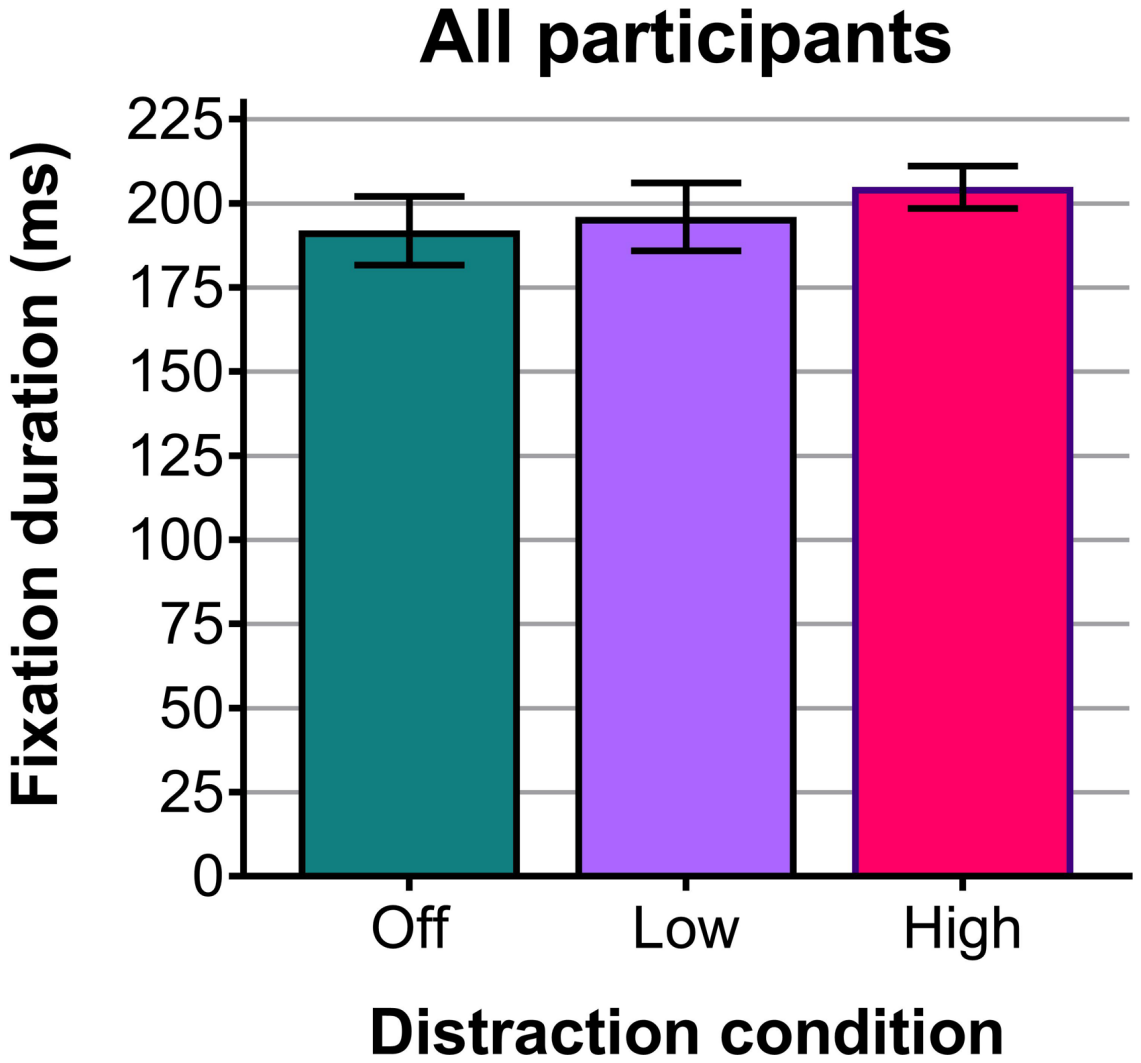
Plot of the mean fixation duration (milliseconds) across all 10 participants for the three distraction conditions. Error bars show the standard errors.

Table 2 shows the eye tracker-derived mean fixation duration for each participant in each distraction condition, together with the overall means which are also plotted in Figure 7 (see Dataset 1, Williamson et al., 2022). Mean fixation durations were longest for the high luminance distraction condition [mean (M) = 205 ms, SD = 20 ms] and shortest for the distraction-off condition (M = 192 ms, SD = 32 ms). Using a one-way repeated measures analysis of variance (ANOVA), the mean fixation durations for distraction condition (Off, Low, High) were found to be statistically significantly different [*F*(2, 18) = 5.318, *p* = 0.015] with a large effect size (partial eta squared = 0.371). Least significant difference *post hoc* tests showed a statistically significant difference between the off and high distraction conditions (13 ms, *p* = 0.023), but not between the off and low distraction conditions (4 ms, *p* = 0.233) or the low and high distraction conditions (9 ms, *p* = 0.061). A Shapiro–Wilk test confirmed normality for each distraction level [W_off_(10) = 0.971, *p* = 0.898, W_low_(10) = 0.971, *p* = 0.899, W_high_(10) = 0.964, *p* = 0.831], while Mauchly’s Test of Sphericity indicated that the assumption of sphericity had not been violated [χ^2^(2) = 1.650, *p* = 0.438].

**Table 2:** Mean fixation duration (milliseconds) for each participant in each distraction condition, together with overall means, standard deviations and standard errors.

	Distraction		
Participant	Off	Low	High
01	255	254	244
02	198	200	205
03	226	224	227
04	160	166	187
05	-	-	-
06	-	-	-
07	186	188	203
08	186	191	194
09	190	187	199
10	205	212	210
11	170	200	208
12	142	136	173
Mean	192	196	205
Standard deviation	32	32	20
Standard error	10	10	6

**Figure 8:**
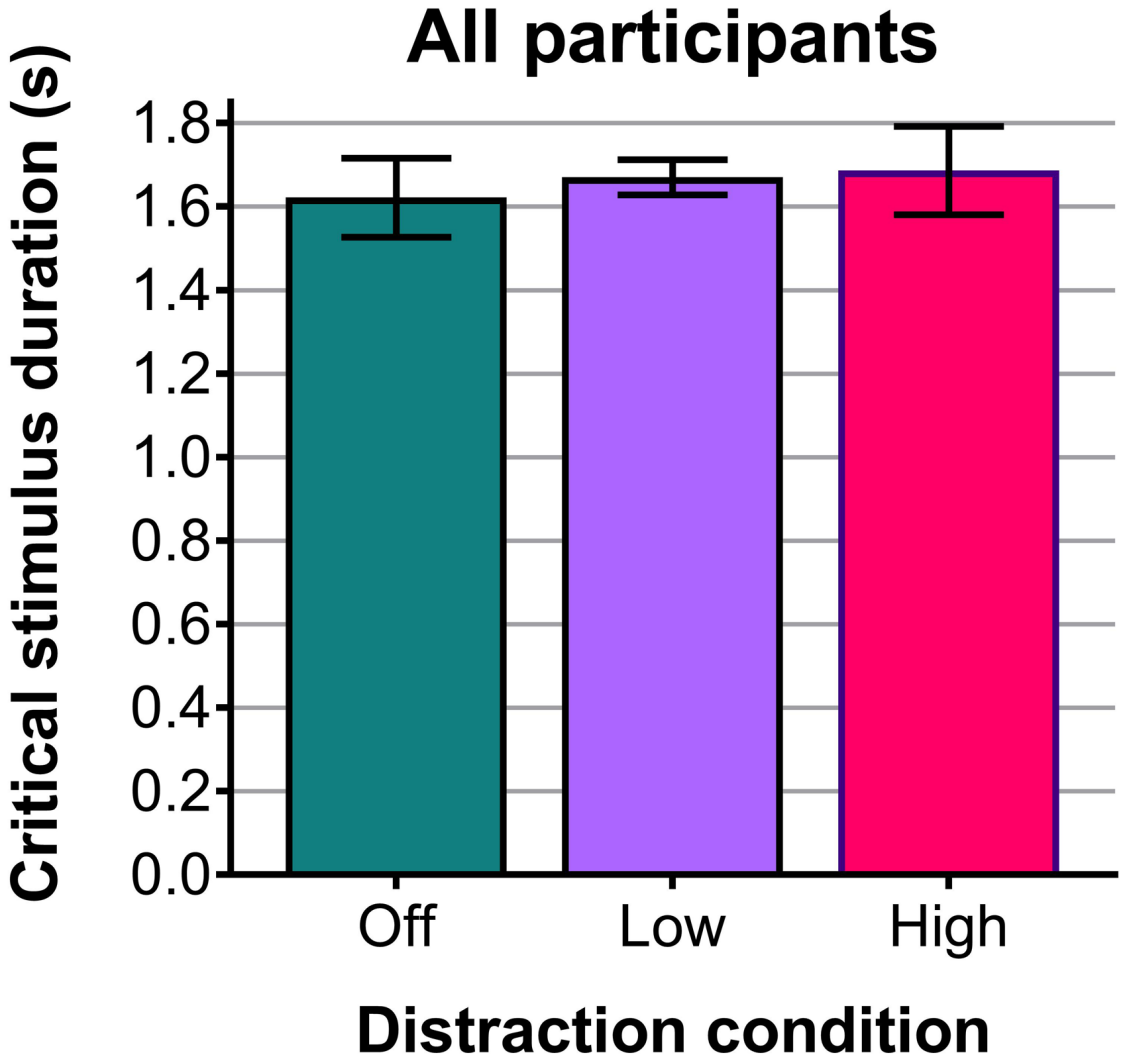
Plot of the mean critical stimulus durations (seconds) across all 12 participants for the three distraction conditions. Error bars show the standard errors.

Table 3 shows the mean critical stimulus duration for each participant in each distraction condition, together with the overall means which are also plotted in Figure 8 (see Dataset 1; Williamson et al., 2022). Mean critical stimulus durations were longest (= worst performance) for the high luminance distraction condition (M = 1.69 s, SD = 0.37 s) and shortest (=best performance) for the distraction-off condition (M = 1.62 s, SD = 0.33 ms). Using a one-way repeated measures ANOVA, the mean critical stimulus durations for distraction condition (off, low, high) were not found to be statistically significantly different [*F*(2, 22) = 0.266, *p* = 0.769]. A Shapiro–Wilk test confirmed normality for each distraction level [W_off_(12) = 0.899, *p* = 0.156, W_low_(12) = 0.910, *p* = 0.211, W_high_(12) = 0.951, *p* = 0.646], while Mauchly’s Test of Sphericity indicated that the assumption of sphericity had not been violated [χ^2^(2) = 0.986, *p* = 0.611].

**Table 3:** Mean critical stimulus durations (seconds) for each participant in each distraction condition, together with overall means, standard deviations and standard errors.

	Distraction		
Participant	Off	Low	High
01	1.04	1.49	0.97
02	2.07	1.87	1.87
03	1.49	1.57	1.74
04	1.81	1.64	2.48
05	1.84	1.77	2.05
06	1.00	1.61	1.57
07	1.75	1.76	1.65
08	1.64	1.47	1.49
09	1.91	1.83	1.42
10	1.69	1.48	1.55
11	1.45	1.74	1.82
12	1.77	1.82	1.63
Mean	1.62	1.67	1.69
Standard deviation	0.33	0.15	0.37
Standard error	0.09	0.04	0.11

Both for mean fixation duration and for critical stimulus duration, three-way ANOVA (distractor x repeat x participant, with participant as a random factor) indicated a significant participant-by-distractor interaction [*F*(18, 97) = 2.452, *p* = 0.003 and *F*(22, 150) = 1.978, *p* = 0.009, respectively], indicating that the effect of the distractor manipulation differed between participants. A secondary analysis was performed at the level of individual participants, using one-way repeated measures ANOVAs to compare between distraction conditions. In this case, repeated measures were over the eight repeats of each distraction condition. Since each repeated set of three trials contained one trial of each distraction condition, data were labeled by set number. For each metric, and each participant, the ANOVA was then used to test for consistent differences between conditions, accounting for differences between sets that may have been introduced by any learning or fatigue effects as the experiment progressed.

Participants 04 and 12 showed a significant effect for fixation duration, while participants 04, 06 and 09 showed a significant effect for critical stimulus duration. Fixation duration effects for participant 04 [*F*(2,12) = 13.411, *p* = 0.001] and participant 12 [*F*(2,12) = 5.906, *p* = 0.016], agreed with the overall trend by showing a significant effect between the off and high luminance distraction conditions, with their fixation durations being longest for the high distraction condition. Additionally, both showed a statistically significant difference between the low and high distraction conditions. For critical stimulus duration, the significant effects for distraction condition varied between participant 04 [*F*(2,14) = 13.704, *p* = 0.001], participant 06 [*F*(2,12) = 11.232, *p* = 0.002], and participant 09 [*F*(2,14) = 3.799, *p* = 0.048]. Participants 04, 06, and 09 all had statistically significant differences between the off and high conditions. However, participants 04 and 06 had a longer critical stimulus duration for the high distraction condition, whereas participant 09 had a longer critical stimulus duration for the distraction-off condition.

The main results were confirmed with linear mixed-effects model analyses, accounting for random effects of participant, which showed significant group-level effects of distraction on fixation duration but not on critical stimulus duration.

## 4. Discussion

In this experiment, bright-light distractions during a combined central and peripheral visual task have been found to cause a statistically significant increase in mean fixation duration (representative of information processing time), rising from 192 ms for the distraction-off condition to 205 ms for the high-luminance distraction condition (*p* = 0.023). This confirmed one of the original hypotheses of this study; that bright-light distractions would increase fixation durations. The results followed the trend anticipated from the literature, with longer durations when exposed to bright-light distractions indicating a decrease in visibility of the low contrast targets or an increase in cognitive workload that required greater processing time for each fixation (Tole et al., 1982; Irwin, 2004). The range of mean fixation durations in this study (192 to 205 ms) falls within the typical range for visual search tasks of 180 to 275 ms given by Rayner (2009).

Only the high-luminance distraction had a significant effect on fixation duration compared to distraction-off, indicating that the presence of transient events at the scene luminance (as in the low luminance distraction condition) is not sufficient “distraction” to significantly alter fixation duration. This suggests that the influence of peripheral distractions on fixation duration might be usefully parameterized as a function of distraction luminance in future research. In an applied setting, such as road safety, it would suggest that the luminance of bright-light distractions, such as on-coming car headlights, may directly affect information processing impairments.

Although the eye-tracking metric revealed an effect of bright-light distraction, the global measure of task performance—the critical stimulus duration for successful completion of the central and peripheral visual tasks—was not significantly affected by the distraction manipulations in this experiment. This is in contrast to the laser-based USAFRL PEDLS (McLin et al., 2019) experiment, which did show a statistically significant decrement in performance with distractions for some conditions (with the same numbers of participants and the same numbers of repeats as our study, and with an identical task). The closest matched condition in the PEDLS experiment (mountain scene, aircraft targets, 10 cd∙m^−2^ average scene luminance), showed a statistically significant difference in critical stimulus duration for distraction condition [Wilks’ Lambda = 0.371, *F*(1,11) = 18.639, *p* = 0.001, multivariate partial eta squared = 0.629]. The difference in critical stimulus durations between the distraction-off (M = 1.54 s, SD = 0.28 s) and with-distraction (M = 1.83 s, SD = 0.33 s) conditions was 0.29 s.

Importantly, PEDLS included the effects of laser eye dazzle as well as distraction. The PEDLS laser source delivered an irradiance of 60 μW∙cm^−2^ at the cornea which is equivalent to an illuminance of around 360 lux. This compares to the maximum 9,000 cd∙m^−2^ luminance of the distraction from the HDR display that was measured as an illuminance of 30 lux at the cornea; more than an order of magnitude lower than PEDLS. At the high illuminance levels of a laser strike, although a laser beam typically has a small angular size, it produces a visual dazzle field of much larger size, which obscures other visual stimuli. A critical difference between the present experiment and PEDLS is the distraction obscuration size. Indeed, the laser irradiance of PEDLS is predicted to cause a visual dazzle field of around 14° full angle at an ambient luminance of 10 cd∙m^−2^ (Williamson and McLin, 2015, 2018), which is equivalent to a distractor size of around 1,160 pixels in the HDR setup. As the present experiment sought to discover the impact of bright-light distractions, rather than dazzle fields, pilot testing for this experiment led to the selection of a 300 pixel distractor size, equivalent to a 3.6° full angle.

Taking these results together, we might usefully separate different regimes of bright-light distraction. Bright-light distractions (the “high” condition in the present experiment) produce more impairment than distraction events that are matched in luminance to the background; and lights that additionally produce a visual dazzle field are have a greater impact still.

Motivated by previous reports of large individual differences in susceptibility to distraction in visual search tasks, and by significant distractor by participant interactions in our analyses, we performed a secondary analysis at the level of individual participants. Two participants (of 10) showed reliable effects of distraction condition on mean fixation duration, and three participants (of 12) showed reliable effects for critical stimulus duration. Using a critical *value of p* of 0.05, the Type 1 error rate is one-in-twenty, so these results suggest that there are individuals in our sample for whom performance is affected by the distraction manipulation.

There are a multitude of factors that could affect susceptibility to distraction, and potential individual differences in the inter-play of these factors. Limitations of this study included the age-diversity of participants, the use of a single set of images, and the simplified task demands compared to real-world scenarios. The age of the volunteer cohort (mean age of 20.2 ± 0.7) was not representative of the population. Older cohorts would be expected to have greater dispersion in executive functions and processing speed (Damoiseaux et al., 2008), as well as differences in attentional capture (Pratt and Bellomo, 1999). It is also possible that the number of peripheral targets and/or their color and contrast differences may affect differences in performance caused by visual distraction (Nagy and Sanchez, 1992; Wolfe, 1994). Furthermore, while saccadic inhibition and inhibition of return were identified as two potential causes of a performance decrement, there are other mechanisms that could have enhanced performance with the distractions. Concepts such as exogenous attention (Theeuwes, 1991), enhanced attention prior to distractors (Makovski, 2019), the attentional boost effect (Swallow and Jiang, 2010), and reduction of attentional blink (Arend et al., 2006), could have contributed in this experiment.

This study has provided new insight to the mechanisms by which bright-light distractions could impact transportation safety. Prior research used laser distractions that introduced the confounding effects of laser eye dazzle. This study used a bespoke MPHDR display to avoid these effects while still providing high luminance distractions. This study also furthered the existing bright-light distraction literature by introducing eye tracking to understand how distraction events translate into changes in information processing times. Ultimately, more research is needed to understand the combined sensory, perceptual, and cognitive factors that influence the effect of bright-light distractions on performance, and individual differences in susceptibility to these effects. The sensitivity of the eye-tracking metric suggests that it might be a useful measure to help gain mechanistic insight in future studies.

While motivated by the importance of understanding bright-light distractions to improve transportation safety, the present experiment was not representative of a real-world transportation task, and therefore it is not possible to conclude whether the results indicate a measurable impact on transportation safety. Future experiments should look to replicate driving and/or piloting tasks and employ bright-light distractions based on real-world data. The use of driving or flight simulators would enable a more realistic environment for these experiments, while allowing a wider range of performance impacts to be assessed such as hazard identification, reaction times, and cognitive workload. The realism of bright-light engagements could be strengthened through the use of devices such as laser event recorders that gather data from actual laser incidents (Williamson and McEwan, 2012; Tipper et al., 2019). These devices could inform the location and duration of laser strikes, together with the encountered laser wavelengths and irradiances. Subsequently, these could be replicated in a laboratory environment, potentially with lasers and/or other means such as high dynamic range displays capable of delivering sufficient illuminance. Such experiments could give a clearer indication of the impact of bright-light distractions and dazzle on car drivers and aircraft pilots.

## Data availability statement

The datasets presented in this study can be found in online repositories. The names of the repository/repositories and accession number(s) can be found at: figshare, https://dx.doi.org/10.6084/m9.figshare.19525567.

## Ethics statement

The studies involving human participants were reviewed and approved by the University of Oxford Central University Research Ethics Committee (CUREC) and the Ministry of Defence Research Ethics Committee (MODREC). The patients/participants provided their written informed consent to participate in this study. Written informed consent was obtained from the individual(s) for the publication of any potentially identifiable images or data included in this article.

## Author contributions

CW and HS developed the methodology. CW coded the experiment. JM collected experimental data. CW, JM, and HS analyzed the data and contributed to the manuscript. All authors contributed to the article and approved the submitted version.

## Funding

This research was jointly funded between the Defence Science and Technology Laboratory (Dstl, which is part of the Ministry of Defence), the University of Oxford, and the EPSRC (EP/W004534/1).

## Conflict of interest

The authors declare that the research was conducted in the absence of any commercial or financial relationships that could be construed as a potential conflict of interest.

## Publisher’s note

All claims expressed in this article are solely those of the authors and do not necessarily represent those of their affiliated organizations, or those of the publisher, the editors and the reviewers. Any product that may be evaluated in this article, or claim that may be made by its manufacturer, is not guaranteed or endorsed by the publisher.

## Footnotes

^1^Averages taken across the most recent 5  years of available data: United Kingdom military (2013–2017); United Kingdom civilian (2014–2018); United States civilian (2017–2021).
